# Bacterial Degradation of Petroleum Hydrocarbons in Saudi Arabia

**DOI:** 10.3390/toxics12110800

**Published:** 2024-11-04

**Authors:** James Mordecai, Assad Al-Thukair, Musa M. Musa, Irshad Ahmad, Alexis Nzila

**Affiliations:** 1 Department of Bioengineering, King Fahd University of Petroleum and Minerals, Dhahran 31261, Saudi Arabiathukair@kfupm.edu.sa (A.A.-T.); irshad@kfupm.edu.sa (I.A.); 2 Department of Chemistry, King Fahd University of Petroleum and Minerals, Dhahran 31261, Saudi Arabia; musam@kfupm.edu.sa; 3 Interdisciplinary Research Center for Refining and Advanced Chemicals, King Fahd University of Petroleum and Minerals, Dhahran 31261, Saudi Arabia; 4 Interdisciplinary Research Center for Membranes and Water Security, King Fahd University of Petroleum and Minerals, Dhahran 31261, Saudi Arabia

**Keywords:** petroleum pollutants, biodegradation, bacteria, aromatics, polyaromatic hydrocarbons, metabolites

## Abstract

The Kingdom of Saudi Arabia (KSA) is the leading oil-exploring and -exploiting country in the world. As a result, contamination of the environment by petroleum products (mainly hydrocarbons) is common, necessitating strategies for their removal from the environment. Much work has been conducted on bacterial degradation of hydrocarbons in the KSA. This review comprehensively analyzed 43 research investigation articles on bacterial hydrocarbon degradation, mainly polyaromatic hydrocarbons (PAHs) within the KSA. More than 30 different bacterial genera were identified that were capable of degrading simple and complex PAHs, including benzo[a]pyrene and coronene. Different strategies for selecting and isolating these bacterial strains and their advantages and disadvantages were highlighted. The review also discussed the origins of sample inocula and the contributions of various research groups to this field. PAH metabolites produced by these bacteria were presented, and biochemical pathways of PAH degradation were proposed. More importantly, research gaps that could enrich our understanding of petroleum product biodegradation mechanisms were highlighted. Overall, the information presented in this paper will serve as a baseline for further research on optimizing bioremediation strategies in all petroleum-contaminated environments.

## 1. Introduction

Fossil fuels, encompassing crude oil, petroleum, coal, and natural gas, have historically been the world’s primary industrial energy production source. Furthermore, numerous crucial industrial products stem from these fossil fuels, including saturates, aromatics, bitumen, asphalt, and asphaltenes [1]. Derived from buried and fossilized remains of ancient plants and animals, fossil fuels primarily consist of hydrocarbons, compounds composed predominantly of hydrogen and carbon atoms. The high demand for energy and the aforementioned products has led to the development of major industries, consequently causing widespread environmental contamination. This contamination often occurs through oil or gas leakage during exploitation and transportation, oil spills resulting from accidents, and the release of by-products, such as plastic [2,3].

Crude oil is a complex mixture comprising various hydrocarbons, including alkanes or saturates, aromatics, resins, and asphaltenes [4]. Aromatics encompass mono-aromatic hydrocarbons (MAHs), such as phenol and its derivatives, as well as polycyclic aromatic hydrocarbons (PAHs) with two or more rings, such as naphthalene (two fused rings), phenanthrene (three fused rings), pyrene (four fused rings), and benzo[a]pyrene and benzo[k]fluoranthene (five fused rings), along with coronene (seven fused rings). Resins and asphaltenes are more complex compounds with higher molecular masses than saturates and aromatics. They comprise aromatic and non-aromatic rings, with small hydrocarbon side chains and occasionally one or more heteroatom(s) [4]. 

Alkanes or saturates are comparatively easier to degrade, followed by mono-aromatic hydrocarbons (MAHs), and then polycyclic aromatic hydrocarbons (PAHs), with the more complex PAHs posing greater challenges in degradation, thus contributing to their accumulation in the environment [5,6,7]. The toxicity of hydrocarbons in general, particularly PAHs, is well-documented. These compounds are carcinogenic, with some forming DNA adducts, leading to mutations, following transformation in animals [8,9]. Negative effects on development, reproduction, immune function, and endocrine systems have also been observed in experimental animals exposed to hydrocarbons [10]. Additionally, hydrocarbons have been found to bioaccumulate in plants and aquatic animals, resulting in contamination and disruption of entire ecosystems [11]. Thus, their removal from the environment remains a priority. 

Several strategies for removing these pollutants have been proposed and evaluated; however, biodegradation (or bioremediation), which relies on the use of microorganisms, remains the best option since it is environmentally friendly and cost effective, compared to physical or chemical approaches [12,13]. In this regard, the literature is replete with work on the isolation and characterization of hydrocarbon-degrading bacteria (HDB), including those that degrade alkanes or saturates, MAHs, and PAHs, from various locations in the world where hydrocarbon contamination is common, and these HDB belong to diverse microbial genera [4,6,7,12,13,14]. Some HDBs have been developed as commercial products for the bioremediation of environments following accidental oil spills on land or sea [5].

The Kingdom of Saudi Arabia (KSA) possesses one of the world’s largest oil industries, with substantial oil reserves and a daily exploitation rate of one million barrels, positioning it as the foremost oil economy globally. Consequently, environmental contamination by oil or its derivatives is prevalent in the KSA [15,16]. For instance, the extraction of every one liter of oil generates three liters of “produced water” (PW), a byproduct of reservoir flushing, resulting in a staggering 30 million barrels of PW produced daily [3,17]. Although a majority of PW, which also contains petroleum products, is recycled, approximately 10–20% is discharged into the environment [3,17]. This, coupled with oil spills from leaks and accidents during extraction, refining, and transportation, as well as emissions from oil combustion, poses significant environmental challenges in the KSA.

As previously discussed, biodegradation or bioremediation represents the most effective and sustainable approach to removing these pollutants from the environment. In pursuit of this objective, extensive research has been conducted in the KSA, focusing on isolating and characterizing hydrocarbon-degrading bacteria (HDB). Various species of HDB have been identified, with some demonstrating the ability to degrade oil products in extremophilic conditions. To provide deeper insights into these HDB and their capacity to degrade oil products, we conducted a systematic literature review of research conducted on HDB in the KSA. This review encompasses (i) a summary of the nature of HDB and the extent of their distribution, (ii) an overview of the hydrocarbon compounds they degrade, (iii) a discussion of the biochemical mechanisms involved in hydrocarbon degradation, and (iv) an exploration of the unique characteristics of these HDB. Additionally, this review identifies and discusses research gaps that need to be addressed to enhance our understanding of PAH bioremediation, in light of the extensive research conducted globally on bioremediation

## 2. Approach to Data Collections

Published and peer-reviewed articles were systematically collected through an exhaustive search across five databases: PubMed (https://pubmed.ncbi.nlm.nih.gov, accessed on 20 July 2024), Scopus (https://www.scopus.com, accessed on 22 July 2024), ScienceDirect (https://www.sciencedirect.com, accessed on 22 August 2024), Web of Science (https://clarivate.libguides.com, accessed on 20 June 2024), and Google Scholar (https://scholar.google.com, accessed on 23 July 2024). The search utilized various combinations of keywords, including bacteria, degradation, hydrocarbons, and Saudi Arabia. Articles meeting the following four inclusion criteria were selected for further analysis: (i) original articles conducted in Saudi Arabia (the location where samples were collected) focusing on crude oil, petroleum, n-alkanes, BTEXs (benzene, toluene, ethyl-benzene, and xylene), monoaromatic hydrocarbons, PAHs, diesel, or kerosene (as degraded pollutants), (ii) featuring bacteria as the sole microorganism, (iii) published between 1983 and 2024, and (iv) written in English. Studies involving fungi, microalgae, or plants in hydrocarbon degradation were excluded, as were studies on bacterial degradation of hydrocarbons using samples not collected in Saudi Arabia. All data were meticulously recorded in a table using Microsoft^®^ Excel 4.3.4.28, and diagrams and graphs were generated using the same Microsoft^®^ Excel software.

**Figure 1:**
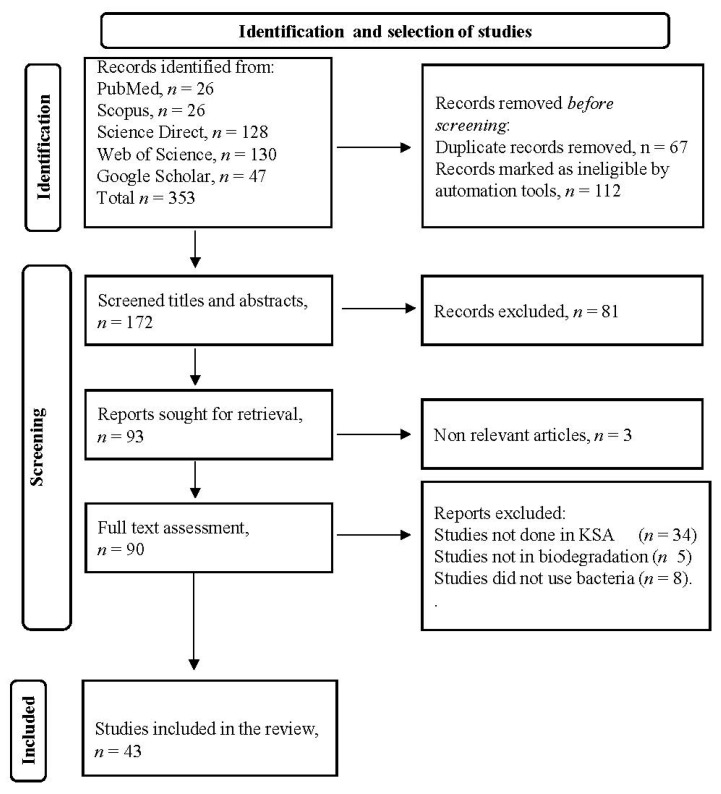
Flow chart showing the selection process of the research articles used in this review.

The utilization of the aforementioned inclusion criteria resulted in the identification of 353 publications. After removing duplicated articles and applying eligibility criteria, a final set of 43 publications was obtained for thorough examination. These selected publications formed the basis of this review (Figure 1).

## 3. General Findings

### 3.1. Frequency of Publications, Research Institutions, and Site of Sample Collections

**Figure 2:**
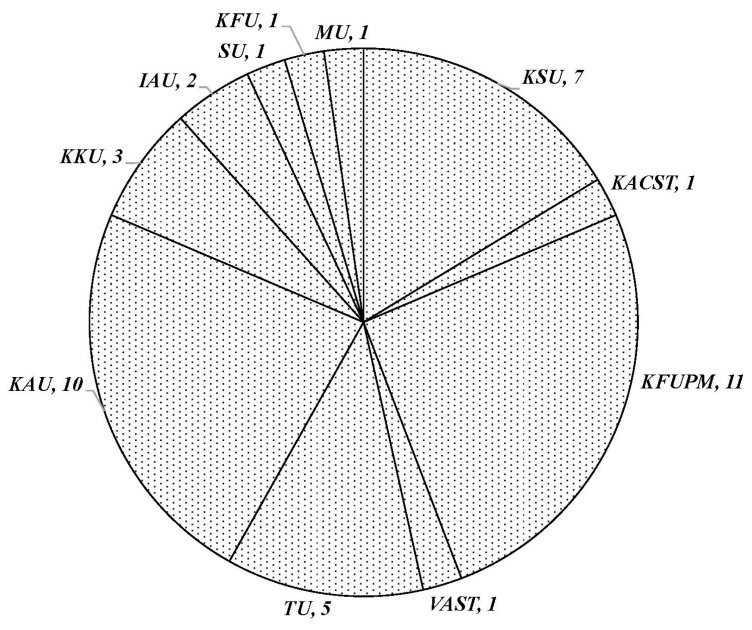
Distribution of studies as a function of the research institutions or universities. The numbers of studies are also indicated. Imam Abdul Rahman bin Faisal University (IAU); King Abdulaziz City for Science and Technology (KACST); King Abdulaziz University (KAU); King Faisal University (KFU); King Fahd University of Petroleum and Minerals (KFUPM); King Khalid University (KKU); King Saud University (KSU); Majmaah University (MU); Shaqra University (SU); Taif University (TU); Vietnam Academy of Science and Technology (VAST).

Research was conducted in nine institutions, including research centers and universities. Notably, King Fahd University of Petroleum and Minerals (KFUPM), King Abdulaziz University (KAU), and King Saud University (KSU) collectively contribute to over 65% of the reported studies. These universities are situated in the three major cities across three provinces in the KSA: KSU in Riyadh, Riyadh Province; KFUPM in Dhahran, Eastern Province; and KAU in Jeddah, Makkah Province (Figure 2).

**Figure 3:**
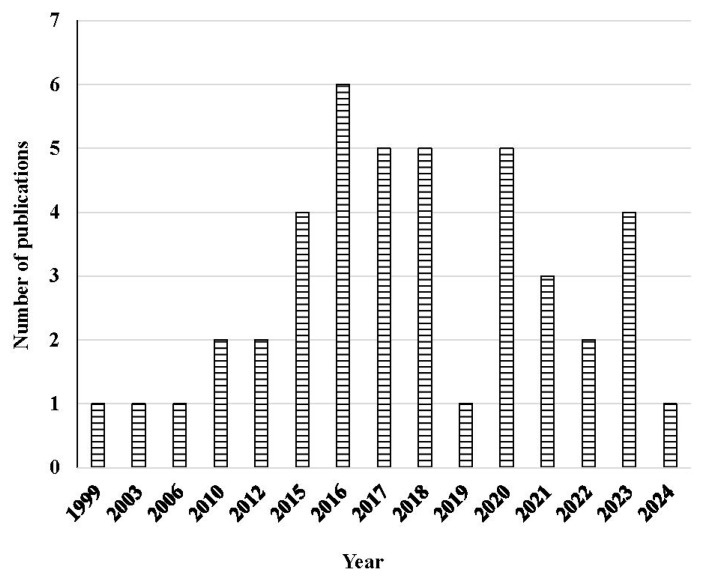
Number of publications as a function of year.

The initial reported work on the biodegradation of hydrocarbons in the KSA dates back to the 1990s, with the first study meeting our inclusion criteria in 1999 [18]. Initially, an average of 1–2 publications were reported each year until 2014. However, from 2015 onwards, the annual number increased from 2 to 7, with an approximate average of 3 per year (Figure 3). 

The onset of studies in the 1990s in the KSA may not be coincidental. In the year 1991, during the Gulf War, a substantial quantity of crude oil was released into the Arabian Gulf, impacting the entire eastern coastline of the KSA. Estimates suggest that between 4 to 10 million barrels of oil were released, forming a slick measuring 101 by 68 km and penetrating up to 40 cm into the sand and mud flats of Saudi Arabia’s shorelines, marking it the largest oil spill in history [15]. Following the war, the KSA initiated numerous studies to investigate the short and long-term impacts of this pollution on the environment [15].

This prompted the exploration and characterization of bacteria capable of degrading petroleum products, which could be utilized in bioremediation strategies. This could potentially account for the increased number of publications from the 1990s onwards.

**Figure 4:**
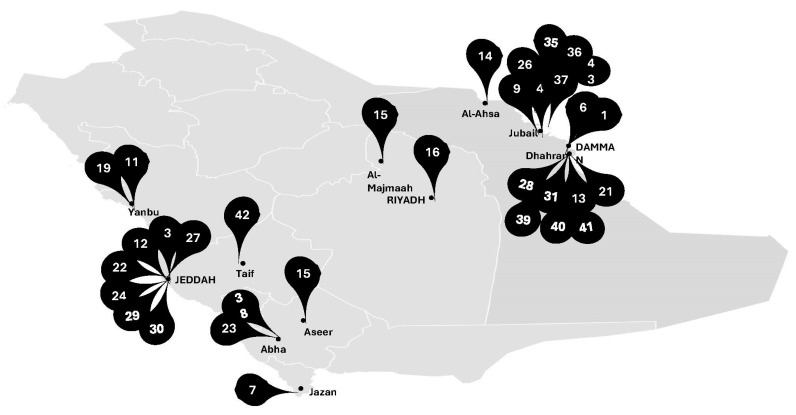
Location of samples used in the studies on bacterial biodegradation of petroleum products in the Kingdom of Saudi Arabia (KSA). The number represents the “study number” as detailed in Table 1.

Supporting this hypothesis, most samples or inocula were collected in the Eastern Province of the KSA, the region most affected by the 1991 oil spill in the Arabian Gulf (Figure 4). Additionally, the Arabian Gulf is recognized as the world’s largest oil reserve [16].

### 3.2. Bacterial Strain Identification

**Figure 5:**
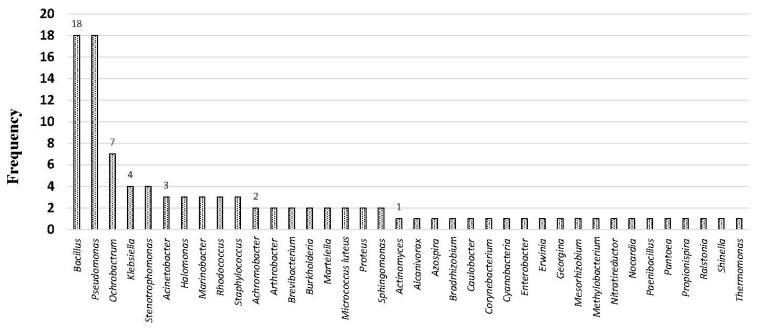
Frequency of bacterial strains per genus from a total of 102 identified strains.

Figure 5 presents a summary of the frequency of identified bacterial genera. In total, 109 bacterial strains were identified. Among these, eighteen strains were attributed to the genera *Bacillus* and *Pseudomonas*. Additionally, seven strains were classified under the genus *Ochrobactrum*, while four strains belonged to *Stenotrophomonas* and *Klebsiella* genera. Furthermore, three strains were associated with each genera *Acinetobacter, Halomonas, Marinobacter, Rhodococcus, and Staphylococcus*. Genera such as *Achromobacter, Arthrobacter, Burkholderia, Martelella, Micrococcus, Proteus,* and *Sphingomonas* each comprised two strains. Notably, bacterial strains belonging to 20 different genera were identified only once (Figure 5).

Among the seventeen bacterial strains within the genus *Pseudomonas*, six were identified as *Pseudomonas* sp., indicating an inability to assign them to a specific species. Notably, the most prevalent species was *Pseudomonas aeruginosa*, with five strains identified, followed by *Pseudomonas citronellolis* and *Pseudomonas stutzeri*, each represented by two strains. Concerning the genus *Bacillus,* out of the eighteen strains, *Bacillus subtilis* and *Bacillus cereus* emerged as the dominant species, each with three strains. In comparison, three strains were categorized as *Bacillus* sp. The remaining nine strains were each associated with a distinct species (Table S1).

The strains attributed to the *Pseudomonas* and *Bacillus* genera stand out as the most predominant in the degradation of petroleum hydrocarbons in the KSA. *Pseudomonas* species are renowned for their remarkable biochemical versatility, particularly in hydrocarbon degradation, and have been extensively documented in the remediation of diverse contaminated environments [19]. The initial evidence showcasing the capability of *Pseudomonas* bacteria to degrade petroleum products dates back a century ago [20]. Since then, significant research efforts have elucidated their capacity to degrade a wide range of compounds, including alkanes, aromatics [19,21,22], chloroaromatics [23], synthetic plastics and pesticides [24,25]. Similarly, the genus *Bacillus* is recognized for its biochemical versatility, with numerous studies highlighting its proficiency in degrading various pollutants, including hydrocarbons (monoaromatics and polyaromatics), crude oil waste, and plastics, among others [26].

One notable attribute contributing to the biodegradative properties of *Bacillus* and *Pseudomonas* is their ability to express and release biosurfactants. These molecules enhance the solubility of pollutants, facilitating their absorption and subsequent degradation [27,28,29]. Intriguingly, a similar study involving a systematic review of bacterial hydrocarbon degradation in Colombia also underscored the predominance of the *Pseudomonas* and *Bacillus* genera [29]. This pattern may be a recurrent feature in various oil-contaminated environments.

### 3.3. In Vitro Culturing of the Bacterial Strains

The rationale of most studies discussed in this review was the search for active bacterial strains suitable for bioremediation. To this end, two methods of bacterial isolation are generally used. The “enrichment protocol” in a selective medium and the “prior growth” in a rich medium or in a non-selective medium. Both approaches require prior in vitro culturing of the bacterial strains, a process that is known to reduce bacterial diversity since only a tiny proportion of bacterial strains, less than 1%, are cultivable in vitro [30,31]. However, despite this common limitation, each approach has advantages and disadvantages. 

In the “enrichment protocol”, strains in the inoculum (contaminated samples) are cultured in a medium containing the pollutant of interest as the sole carbon source. This progressively leads to the enrichment of bacteria strains capable of degrading the pollutant after several culture transfers or passages. Bacterial stains that cannot utilize the selected carbon source will be eliminated because their growth will be lower or null, while those that prefer the selected carbon source will be favored in the culture, leading to their isolation. At the end of the enrichment process, only active strains degrading the selected pollutant as a source of carbon will be isolated [32]. 

In the second approach, the “prior growth” in rich medium, strains from contaminated samples are first grown in a rich medium, a non-selective medium, which permits the growth of all microorganisms, so long as they can grow in vitro. Thereafter, each strain is isolated and its ability to individually degrade the pollutant of interest is evaluated. As a result, not all isolated bacterial strains (from the initial culture in a rich medium) will be active in the degradation of the pollutant of interest. 

Additionally, in the initial culture (in rich medium), active strains can outcompete those that can degrade the pollutant of interest (since the culture is not selective), leading to low efficiency in selecting pollutant-degrading bacterial strains. Consequently, an “enrichment protocol” is commonly used for the selection and isolation of active bacterial strains degrading a specific pollutant [32]. In support of this, more than 75% of bacterial strains reported in this review were derived from an enrichment protocol (ST10–ST41) [18,33,34,35,36,37,38,39,40,41,42,43,44,45,46,47,48,49,50,51,52,53,54,55,56,57,58,59,60,61,62,63,64,65], while “prior growth” in the rich medium was employed in only seven studies (ST1–ST3, ST6–ST9) [66,67,68,69,70,71,72] In three studies, the protocol of bacterial isolation was not mentioned (ST5, ST42–ST43) [62,64,73]. 

As mentioned earlier, “prior growth” in a rich medium leads to the selection of many strains, and some may not necessarily be active in pollutant degradation. This is illustrated in ST7 [70], in which a total of 43 strains were isolated, and out of these, only six, belonging to the genera *Pseudomonas, Bacillus, Staphylococcus, Erwinia,* and *Nitratireductor,* were active in degrading a crude oil product [70]. Thus, unlike “the enrichment protocol”, “prior growth” in the rich medium does not necessarily guarantee that the initially selected strains (after the culture in the rich medium) will be active to degrade a given pollutant. 

In addition to the culturing methods, non-culturing strategies can be employed to study the degradation of petroleum products. They involved the identification of bacteria strains using various molecular techniques such as denaturing gradient gel electrophoresis (DGGE), restriction fragment length polymorphism (RFLP), 16S rRNA sequencing, or whole genome analysis [74]. This approach provides information on non-culturable and culturable bacterial strains in their natural environment, without any prior selection. As a result, it does not allow the isolation of bacterial strains and, thus, has limited interest in bioremediation strategy. This approach identifies bacterial communities in given samples but does not provide information on the ability of each of these strains to degrade pollutants. 

Several reports have been dedicated to the use of this approach to study crude oil degradation [75]; however, so far, only one study has employed this approach in Saudi Arabia. In this study, the DGGE approach was used to identify the existing bacteria in contaminated samples, and the results showed very diverse genera, including *Thiobacillus, Thiobaca, Halochromatium, Pseudomonas, Alcanivorax, Deinococcus, Desulfosarcina, Cytophaga, Holophaga, Acidobacteria, Geothrix, Verrumicrobia, Chloroflexus Spirochetes*, and *Planctomycetes*, among others [76]. The identification of diverse genera is a common feature of this approach since there is no prior in vitro bacterial culturing [74,75,77]. However, as mentioned before, the contribution of each of these strains to the degradation cannot be established since none of the strains was cultured; thus, their potential for bioremediation cannot be evaluated. 

### 3.4. Substrates Used in Studies: Crude Oil, Aliphatic, and Polyaromatic Compounds

As discussed earlier, in the “Prior Growth protocol” using a rich medium, bacteria are isolated first, and then their ability to degrade a given substrate is evaluated afterward. However, in the “Enrichment Protocol”, the selection of bacterial strains is carried out in the presence of a specific substrate, ensuring that the resulting bacterial strains will actively degrade this particular substrate. Subsequently, their ability to degrade other substrates can be evaluated.

Strains isolated using the “Prior Growth protocol” are not always associated with the degradation of the substrate of interest. However, in the KSA, some of these strains have been associated with the degradation of crude oil; the aliphatic compounds n-hexadecane, pristane, and n-octadecane; the monocyclic aromatic hydrocarbons (MAHs) phenol and benzene; and the polycyclic aromatic hydrocarbons (PAHs) phenanthrene, fluoranthene, pyrene, and dibenzothiophene (Table 1, ST1-ST9).

**Figure 6:**
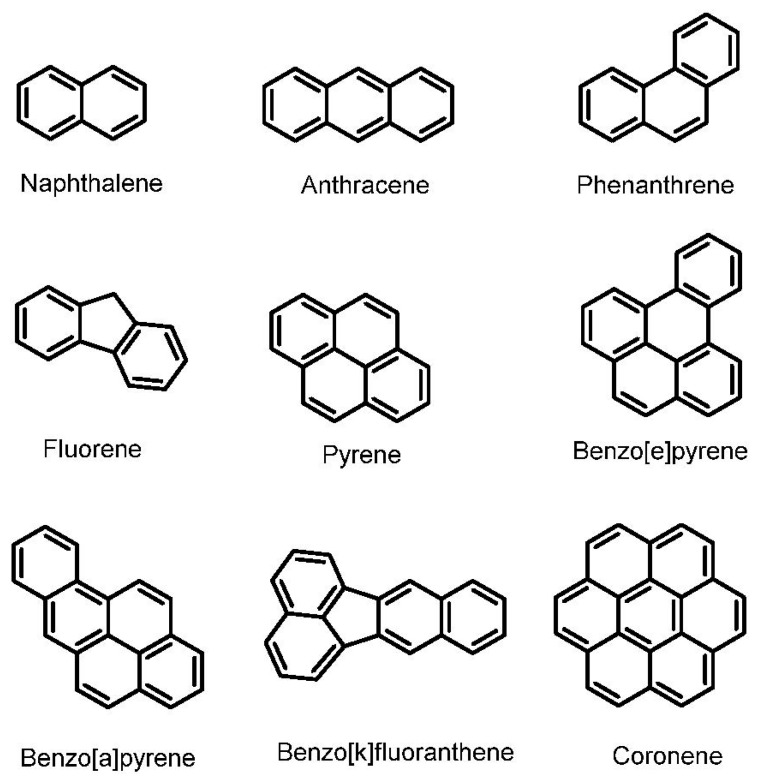
Chemical structures of PAHs discussed in this study.

In contrast, in the “Enrichment Protocol”, substrates that needs to be degraded are used during the enrichment process, leading to the isolation of bacterial strain that selectively degrade these substrates. As shown in Table 1, the initial substrates used included: crude oil (ST10–ST14); diesel (ST15); the aliphatics hexadecane, pristane, n-hexadecane, and n-tetradecane (ST16–ST18); the MAHs phenol and BTEX (ST19, ST20); and the PAHs anthracene (ST21, ST22), phenanthrene (ST23–ST30), a mixture of anthracene/phenanthrene or anthracene/phenanthrene/fluorene (ST31–ST34), pyrene (ST35–ST38), benzo(a)pyrene (ST39, ST40), and coronene (ST41). Thus, in order of importance, the substrates used to select active bacteria were PAHs (21 out of 43 studies), followed by crude oil and diesel (6 studies), and MAHs (2 studies). Figure 6 shows the chemical structure of PAHs discussed in this review.

Aliphatic degradation was carried out in 3 studies only (ST16–ST18). As discussed earlier, investigations on aliphatic degradation are of limited interest since these compounds are generally easy to degrade. Thus, they do not accumulate in the environment, which explains why there are fewer studies compared to those on aromatics. In addition, their mechanisms of degradation have already been extensively studied [78].

In the 23 studies where PAHs were used, phenanthrene alone was employed in 11 studies (ST23–ST34), and phenanthrene was used in combination with anthracene in 3 studies (ST32–ST34). In comparison, only two studies used anthracene as the sole carbon source (ST21, ST22). The four-ring PAH pyrene was employed in 4 studies (ST35–ST38) and the five rings benzo[a]pyrene in two studies (ST39, ST40), and finally, only one study was undertaken with the use of coronene (ST41). Thus, it is clear that phenanthrene was the most used PAH, followed by pyrene, and limited work has been carried out on benzo[a]pyrene and coronene. These observations are, overall, in line with the many studies carried out on the degradation of PAHs all over the world [5,6,7,79,80,81,82]. The PAH coronene is one the most recalcitrant PAHs, and so far, its biodegradation has been reported in only one other study [83].

Crude oil was used as an enrichment substrate in 5 studies (ST10–ST14), while diesel was employed in one study (ST15). As stated earlier, crude oil consists of various aliphatic and aromatic compounds, and diesel also, to a lesser extent [4,84]. When used as a carbon source, bacteria will degrade components that are more amenable to degradation, mainly the aliphatic ones. Thus, such a mixture of petroleum products in enrichment does not guarantee the selection of bacteria active in degrading complex petroleum products. Supporting this, in 4 studies (ST10–ST13), the isolated bacteria were tested on crude oil only, and only one study reported the degradation of PAHs by bacterial strains isolated under these conditions (using crude oil as an enrichment substrate) [ST14].

**Table 1:** Summary of work published on bacterial isolates collected from the Kingdom of Saudi Arabia. GC and HPLC represent gas chromatography and high performance liquid chromatography respectively.

Study Number (ST #)	Pollutant Used to Isolate Bacterial Strains	Type of Inoculum	Location of Inoculum Collection	Research Center or University	Method of Strain Selection	List of Isolated Bacteria	Other Pollutants Degraded by Bacterial Strains	Type of Bacteria	Main Results	Reference
ST 1	None	Contaminated Soil	Dammam	King Saud University (KSU)	Prior growth but medium not mentioned	*Staphylococcus Corynebacterium*	Crude Oil	Mesophile	30–70% of degradation rate after 28 days, using weighing method	[66]
ST 2	None	Crude Oil Samples	No Information	Taif University (TU)	Prior growth in rich medium	*Klebsiella* *Acinetobacter*	Phenanthrene, Fluoranthene, Pyrene, Benzene	Mesophile	58–83% degradation, after 48 h, by weighing method	[67]
ST 3	None	Petroleum Sludge	Jeddah	Taif University (TU)	Prior growth in wastewater	*Pantoea* *Acinetobacter* *Bacillus*	n-Hexadecane, Phenol, Phenanthrene	Mesophile	Almost 100% degradation within 4 days, GC	[68]
ST 4	None	Crude Oil contaminated Microbial Mats	Dawhat, Al-Daffi, Jubail	King Fahd University of Petroleum and Minerals (KFUPM)	Metagenomic Analysis	Cyanobacterial mat.	Pristane, n-Octadecane, Phenanthrene, Dibenzothiophene	Mesophile	14–25% of degradation, by GC	[76]
ST 5	None	Soil	Not mentioned	Imam Abdul Rahman bin Faisal University (IAU)	Not Specified	*Alcanivorax*	Crude Oil	Mesophile	Up to 73% reduction, by GC	[73]
ST 6	None	Soil	Dammam	Imam Abdul Rahman bin Faisal University (IAU)	Prior growth in rich medium	*Bacillus* *Pseudomonas*	Crude Oil	Mesophile	40–56% of oil degradation after 7 days, by spectrophotometry	[69]
ST 7	None	Contaminated Sediments	ARAMCO, Jazan	King Abdulaziz University (KAU)	Prior growth in Bushnell-Haas medium	*Pseudomonas* *Bacillus* *Staphylococcus* *Erwinia* *Nitratireductor*	Crude Oil	Mesophile	67–77% of oil degradation by after 7 days, by spectrophotometry	[70]
ST8	None	Marine Soil Sediment	Eastern Province	King Saud University (KSU)	Prior growth in rich medium	*Bacillus*	Crude Oil	Mesophile	>90% of degradation after 14 days, by HPLC	[71]
ST 9	None	Bilge Wastewater	Aljubail Port	King Saud University (KSU)	Prior growth in Bushnell-Haas medium	*Acinetobacter* *Klebsiella* *Pseudomonas* *Bacillus* *Brevibacterium*	Crude Oil	Mesophile	90% of reduction of crude within 3 days, by weighing	[72]
ST 10	Crude Oil	Contaminated Soil	Arabian Gulf	King Saud University (KSU)	Enrichment Culture	*Pseudomonas*	Crude Oil	Mesophile	>80% degradation within 10 days, GC	[18]
ST 11	Crude Oil	Sediments	Yanbu	King Abdulaziz University (KAU)	Enrichment Culture	*Pseudomonas* *Nitratireductor*	Crude Oil	Mesophile	65–95% degradation 14 days, by GC	[33]
ST 12	Crude Oil	Contaminated Sediment	Jeddah	Taif University (TU)	Enrichment Culture	*Enterobacter*	Crude Oil	Mesophile	87–97% of degradation after 14 days, by gravimetric methods	[34]
ST 13	Crude Oil	Contaminated Soil	Dhahran	Shaqra University (SU)	Enrichment Culture	*Bacillus* *Pseudomonas*	Crude Oil	Mesophile	Not mentioned	[35]
ST 14	Crude oil	Soil	Al-Ahsa	King Faisal University (KFU)	Enrichment Culture	*Georgina* *Arthrobacter*	Crude Oil, n-alkanes, PAHs (biphenyl, naphthalene, and anthracene)	Mesophile	67% of PAH degradation within 14 days, by GC	[36]
ST 15	Diesel	Contaminated Soil	Al-Majmaah	Majmaah University	Enrichment Culture	*Microbacterium*	Diesel	Mesophile	21–78% of degradation after 5 days, by GC-MS	[37]
ST 16	Hexadecane	Contaminated Soil	Riyadh	King Saud University (KSU)	Enrichment Culture	*Pseudomonas Rhodococcus* *Bacillus*	Hexadecane	Mesophile	100% degradation in 5–9 days, using GC	[38]
ST 17	Pristane and hexadecane	Contaminated Sand Samples	No Information	Vietnam Academy of Science and Technology (VAST)	Enrichment Culture	*Nocardia*	Pristane, various C6-C16 alkanes	Mesophile	Up to 90% within 3 weeks, using GC	[39]
ST 18	n-tetradecane	Contaminated Soil	No Information	Taif University (TU)	Enrichment Culture	*Pseudomonas* *Bacillus*		Mesophile	50–90% within 5 days, by GC	[40]
ST 19	Phenol	Wastewater from Industrial Treatment Plant	Yanbu	King Abdulaziz University (KAU)	Enrichment Culture	*Ochrobactrum Pseudomonas*	Phenol	Mesophile	100% degradation within 4 days by Ultra-Fast Liquid Chromatography	[41]
ST 20	Benzene, Toluene, Ethylbenzene, Xylene s	Contaminated Soil, Water and Oily Sludge	Eastern Region	King Abdulaziz City for Science and Technology (KACST)	Enrichment Culture	*Bacillus* *Paenibacillus Burkholderia* *Proteus*	Benzene, Toluene, Ethylbenzene, Xylene	Mesophile	100% degradation except for Benzene, within 21 days, by HPLC	[42]
ST 21	Naphthalene	Contaminated Soil Sample	Dhahran	King Fahd University of Petroleum and Minerals (KFUPM)	Enrichment Culture	*Methylobacterium* *Pseudomonas*	Naphthalene	Mesophile	Not directly determined. Degradation was assessed by bacteria growth	[43]
ST 22	Anthracene	Marine Water	Abhor, Red Sea, Jeddah	King Saud University (KSU)	Enrichment Culture	*Sphingomonas*	Anthracene	Psychrophile		[44]
ST23	Phenanthrene	Contaminated Soil	Abha	King Khalid University (KKU)	Enrichment Culture	*Sphingomonas Pseudomonas* *Micrococcus* *Arthrobacter Stenotrophomonas* *Kocuria* *Shinella*	Naphthalene, Phenanthrene	Mesophile	Not direct assessment (assessment by bacterial growth)	[45]
ST 24	Phenanthrene	Marine Water	Abhor, Red Sea, Jeddah	King Abdulaziz University (KAU)	Enrichment Culture	*Ochrobactrum Propionispira Martelella* *Bacillus* *Marinobacter* *Azospira*	Phenanthrene, Pyrene, Fluorene, Hexadecane	Halophile	88–100% degradation within 12 days, by HPLC and GC	[46]
ST 25	Phenanthrene	Sediments from Mineral Mining Site	No Information	King Abdulaziz University (KAU)	Enrichment Culture	*Stenotrophomonas*	Anthracene, Phenanthrene, Naphthalene, Fluorene, Pyrene, Benzo(e)pyrene, Benzo(k)fluoranthene	Acidophile	80–95% degradation of PAHs by GC	[47]
ST 26	Phenanthrene	Contaminated Sediment	Jubail	King Fahd University of Petroleum and Minerals (KFUPM)	Enrichment Culture	*Pseudomonas* *Ochrobactrum*	Phenanthrene, Pyrene	Mesophile	62–94% degradation, by GC	[48]
ST 27	Phenanthrene	Brine Water Sample	Jeddah	King Abdulaziz University (KAU)	Enrichment Culture	*Ochrobactrum* *Pseudomonas*	Phenanthrene Naphthalene, Anthracene, Fluorene, Pyrene, Benzo(e)pyrene, Benzo(k)fluoranthene	Halothermophile	81–90% degradation, within 14 days, by GC	[49]
ST 28	Phenanthrene	Drilling Site Sediment	Al-Khobar	King Abdulaziz University (KAU)	Enrichment Culture	*Pseudomonas* *Bacillus*	Naphthalene, Phenanthrene, Fluorene, Anthracene, Pyrene, Benzo(e)pyrene, Benzo(k)fluoranthene	Thermophile (60 °C)	81–95% degradation, by HPLC	[65]
ST 29	Phenanthrene	Saline Seawater and Sediment	Abhor, Red Sea, Jeddah	King Abdulaziz University (KAU)	Enrichment Culture	*Ochrobactrum Stenotrophomonas* *Achromobacter Mesorhizobium*	Phenanthrene, Fluorene, Pyrene	Halophile	50–90% degradation within 12 days, by GC	[50]
ST 30	Phenanthrene	Briny Water and Sediment	Red Sea	King Abdulaziz University (KAU)	Enrichment Culture	*Marinobacter (3 strains)*	Phenanthrene, Pyrene	Halophile	71–90% degradation within 12 days, by GC	[51]
ST 31	Phenanthrene or Anthracene	Contaminated Soil Sample	Dhahran	King Fahd University of Petroleum and Minerals (KFUPM)	Enrichment Culture	*Pseudomonas Stenotrophomonas* *Ralstonia* *Thermomonas*	Anthracene, Phenanthrene	Mesophile	Up to 75% degradation within 15 days, by GC	[58]
ST 32	Phenanthrene or Anthracene	Contaminated Soil	Aseer	King Khalid University (KKU)	Enrichment Culture	*Bacillus* *Ochrobactrum*	Anthracene, Phenanthrene	Mesophile	Assessment by bacterial growth	[53]
ST 33	Phenanthrene and Anthracene	Contaminated Soil	No Information	King Saud University (KSU)	Enrichment Culture	*Halomonas*	Anthracene, Phenanthrene	Halophile	95–100% degradation within 1–2 days, by GC-MS	[55]
ST 34	Fluorene	Drilling Site Sample	No Information	King Abdulaziz University (KAU)	Enrichment Culture	*Ochrobactrum* *Bacillus* *Marinobacter Pseudomonas Martelella* *Stenotrophomonas* *Rhodococcus*	Anthracene, Phenanthrene, Fluorene, Naphthalene, Pyrene, Benzo(a)pyrene, Benzo(e)pyrene, Benzo(k)fluoranthene.	Halo-alkalo-thermophile (60 °C)	55–100% degradation within 16 days	[56]
ST 35	Pyrene	Contaminated Soil	Jubail	King Fahd University of Petroleum and Minerals (KFUPM)	Enrichment Culture	*Burkholderia Caulobacter*	Pyrene	Mesophile	21–59% of degradation within 18 days, by GC	[57]
ST 36	Pyrene	Wastewater Sludge	Jubail	King Fahd University of Petroleum and Minerals (KFUPM)	Enrichment Culture	*Halomonas*	Pyrene, Naphthalene, Anthracene, Phenanthrene,	Halophile	50% degradation within 18 days, by GC	[52]
ST 37	Pyrene	Contaminated Soil	Jubail	King Fahd University of Petroleum and Minerals (KFUPM)	Enrichment Culture	*Achromobacter*	Pyrene, Naphthalene, Anthracene, Phenanthrene	Mesophile	50% degradation within 15 days, by GC	[54]
ST 38	Pyrene	Contaminated Soil	Abha	King Khalid University (KKU)	Enrichment Culture	*Klebsiella*	Pyrene, Naphthalene, Anthracene, Phenanthrene, Phenanthrene	Mesophile	70% degradation after 12 days, by HPLC	[59]
ST 39	Benzo(a)Pyrene.	Contaminated Sediment	Dhahran	King Fahd University of Petroleum and Minerals (KFUPM)	Enrichment Culture	*Staphylococcus*	Benzo(a)pyrene, Pyrene, Naphthalene, Anthracene, Phenanthrene	Halophile	44–80% degradation within 30 days, by GC	[60]
ST 40	Benzo(a)Pyrene.	Contaminated Sediment	Dhahran	King Fahd University of Petroleum and Minerals (KFUPM)	Enrichment Culture	*Bradyrhizobium Micrococcus* *Bacillus*	Benzo(a)pyrene, Pyrene, Naphthalene, Anthracene, Phenanthrene	Mesophile	44–75% within 30 days, by GC	[61]
ST 41	Coronene	Contaminated Sediment	Dhahran	King Fahd University of Petroleum and Minerals (KFUPM)	Enrichment Culture	*Halomonas,*	Coronene, Benzo(a)pyrene, Pyrene, Naphthalene, Anthracene, Phenanthrene	halophilic	48% degradation within 20 days, and 76% within 80 days, by GC	[63]
ST 42	Not mentioned	Sediments from Industrial Area	Taif	Taif University (TU)	Not mentioned	*Bacillus* *Actinomyces* *Pseudomonas*	Crude Oil	Mesophile	52–63% degradation, by gravimetric methods	[64]
ST 43	Not mentioned	Contaminated Sediment	Jubail	King Fahd University of Petroleum and Minerals (KFUPM)	Not mentioned	*Brevibacillus* *Proteus* *Rhodococcus*	Naphthalene, Pyrene.	Mesophile	35–62% within 18 days, by GC	[62]

### 3.5. Mesophilic, Halophilic, and Thermophilic Bacteria

In both the “enrichment protocol” and “prior growth”, bacterial cultures are generally grown at a temperature range of 30–40 °C, low salinity of <4% NaCl, and neutral pH. Bacterial strains selected under these conditions are known as mesophiles [61]. These conditions are considered the most prevalent in most contaminated sites. As a result, most of the reported bacteria-degrading pollutants in general, and petroleum products in particular, have been selected under these mesophilic conditions. The same observation can be made in studies carried out in Saudi Arabia, where more than 71% of reported bacteria are mesophilic (Table 1). 

However, contamination also occurs in extreme environments, primarily characterized by high salinity (for halophilic bacteria) and high temperatures (for thermophilic bacteria). Thermophiles are categorized into three groups based on the optimal temperature ranges for bacterial growth: moderate thermophiles (40–59 °C), extreme thermophiles (60–85 °C), and hyper-thermophiles (>85 °C) [85]. In relation to salinity, moderately halophilic bacteria grow in the range of 3–5% (w/v) NaCl, while extremely halophilic bacteria grow at >15% (w/v) NaCl [86].

Nine studies investigated the degradation of PAHs in halophilic conditions, ranging from 4% to 30% NaCl (ST24, ST27, ST29, ST30, ST33, ST34, ST36, ST39, ST41). Interestingly, in most of these studies, bacterial consortia were used rather than single bacterial strains. For instance, the consortium of *Ochrobactrum halosaudia* AJH1, *O. halosaudia* AJH2, and *P. aeruginosa* AJH3 was shown to degrade phenanthrene, the 3-ring polyaromatic hydrocarbon, from 20% up to 30% NaCl, although the efficiency of degradation decreased significantly at 30% (ST27). Furthermore, this consortium could also degrade phenanthrene at temperatures between 30 and 60 °C, making it a halo-thermophilic bacterial consortium (ST27) [65]. Other studies used two or more bacterial strains (ST24, ST26, ST29, ST30, ST33, ST34). 

The use of consortia shows that salinity conditions reduce or limit bacterial growth. As a result, different strains are used as consortia, each providing a small contribution to the degradation process. This is a common feature of pollutant degradation in extreme conditions, particularly in the context of PAH degradation in high salinity [86]. However, our group reported three studies in which single halophilic bacterial strains were used to degrade PAHs (ST36, ST39, ST41). More specifically, Budiyanto et al. isolated two single bacterial strains, *Halomonas shengliensis* 10PY2B and *Halomonas smyrnensis* 20PY1A, that could each degrade pyrene at 10–20% NaCl (ST36). A strain of *Staphylococcus haemolyticus* 10SBZ1A could degrade the highly complex benzo[a]pyrene at 10% NaCl (ST39), and finally, the degradation of the 7-ring coronene was reported using *Halomonas caseinilytica* (ST41). Thus far, the degradation of benzo[a]pyrene in halophilic conditions has been reported only in another study carried out in India, using a single strain, *Ochrobactrum* sp. VA1, but at a relatively low salinity of 3% NaCl [87]. Thus, these Saudi strains are among the most active reported so far for the degradation of benzo[a]pyrene in a hypersaline condition of 20% NaCl. 

In relation to thermophilicity, three studies investigated the degradation of PAHs in thermophilic conditions (ST27, ST28, ST34). As with halophilic conditions, degradation was observed using consortia, and it occurred at temperatures as high as 60 °C (ST27-28, ST34). The degradation of petroleum products, including aliphatics, MAHs, and PAHs, has been reported in different parts of the world and can occur at temperatures as high as 80 °C [7]. Interestingly, almost 60% of these bacteria belong to the *Geobacillus* genus, and 80% belong to the *Bacillaceae* family [7]. However, in Saudi samples, as shown in Table 1, out of 11 genera identified for thermophilic bacterial strains (ST27, ST28, ST34), no *Geobacillus* was present, and only 2 belonged to the *Bacillaceae* family (*Bacillus* strains in ST27 and ST34). 

### 3.6. Identification of Metabolites, Enzymes, and Biochemical Degradation Pathways of PAHs

Biochemical pathways associated with PAH degradation have been investigated by identifying PHA metabolites and enzymes involved in this biodegradation. This knowledge can be utilized to develop microbial biocatalysts with enhanced degradation capabilities by using genetic engineering and synthetic biology strategies [88,89]. These engineered strains could then be used to enhance pollutant biodegradation as part of the bioremediation strategies.

Numerous studies have focused on the identification of PAH metabolites using gas chromatography and liquid chromatography, coupled with mass spectrometry (GC- and LC-MS), and enzyme detection using various techniques, including whole genome analysis, proteomics, and transcriptomes [81,88].

The biodegradation of PAHs is generally initiated by the action of mono- or di-oxygenase enzymes that hydroxylate a ring in PAHs, leading to ring cleavage and the subsequent formation of hydroxylic or carboxylic derivatives with fewer rings. The aliphatic moieties of these derivatives are then removed, allowing them to enter Krebs cycles for energy generation in the form of ATP (adenosine triphosphate). This process continues until all rings are cleaved [82,90].

In the KSA, 11 investigations have reported the identification of PAH metabolites. Six were carried out at the KFUPM (ST21, ST31, ST36, ST37, ST39, ST40), three at KAU (ST25, ST28, ST29), and two at KSU (ST22, ST33). These studies investigated the metabolites of various PAHs, such as naphthalene, phenanthrene, anthracene, fluorene, pyrene, and benzo[a]pyrene.

**Figure 7:**
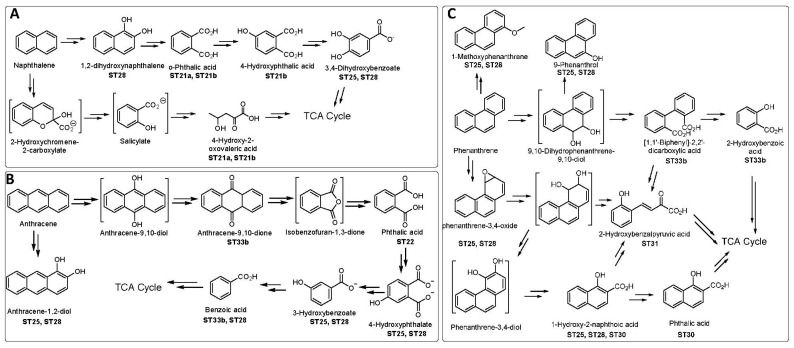
Proposed biodegradation pathways for naphthalene (**A**), anthracene (**B**), and phenanthrene (**C**) by bacterial strains isolated from the Kingdom of Saudi Arabia (KSA). Metabolites in brackets were not detected and are hypothetical. ST21a: *Methylobacterium radiotolerans* N7A0, ST21b: *Pseudomonas aeruginosa* N7B1, ST22: *Sphingomonas* sp. KSU05, ST25: *Stenotrophomonas maltophilia* AJH1. ST28: Consortium of *Pseudomonas aeruginosa* CEES1 and *Bacillus thermosaudia* CEES2. ST30: consortium of *Marinobacter hydrocarbonoclasticus* MAM1, *Marinobacter* sp. MAM2, and *Marinobacter hydrocarbonoclasticus* MAM3; ST31: *Stenotrophomonas maltophilia*. ST33b: *Halomonas* sp. BR04. ST refers to the research study as indicated in Table 1. The small letters refer to a specific strain (for instance, ST21a refers to ST21, but is related to the strain *Methylobacterium radiotolerans N7*).

For instance, the degradation of naphthalene, considered the simplest among PAHs, involves hydroxylation of one of the rings followed by ring cleavage to produce a benzoic acid or benzaldehyde derivative. When *Methylobacterium radiotolerans* N7A and *Pseudomonas aeruginosa* N7B1 (ST21) were used for naphthalene degradation, o-phthalic acid and 4-hydroxy-2-oxovaleeric acid were obtained as metabolites. Furthermore, a consortium of *P. aeruginosa*, *Bacillus thermosaudia*, and *Stenotrophomonas maltophilia* (ST28) produced 1,2-dihydroxynaphthalene and 3,4-dihydroxybenzoate as metabolites in naphthalene degradation. Additionally, 3,4-dihydroxybenzoate was obtained during naphthalene degradation by *St. maltophilia* strain AJH1 (ST25). A tentative metabolic pathway for the biodegradation of naphthalene using bacterial strains isolated from the KSA is shown in Figure 7 (ST21, ST25, ST28). 

The degradation of anthracene and phenanthrene commences with hydroxylation at either the terminal or the inner ring. For instance, anthracene-1,2-diol was observed in the degradation of anthracene using *S. maltophilia* AJH1 (ST25) or a consortium of *P. aeruginosa* CEES1 and *B. thermosaudia* CEES2 (ST28), while anthracene-9,10-dione was obtained in the degradation of anthracene using *Halomonas* sp. BR04 (ST33b). Subsequent ring cleavage of the middle ring of anthracene-9,10-dione led to the generation of o-phthalate derivative, which is further degraded to benzoic acid before entering the TCA cycle. Degradation pathways based on the identified metabolites for anthracene are illustrated in Figure 7 (ST22, ST25, ST28, ST33). Similarly, phenanthrene degradation using various strains isolated in the KSA revealed both oxidations of the middle ring and side rings (ST25, ST28, ST30, ST31, ST33b), as depicted in Figure 7.

**Figure 8:**
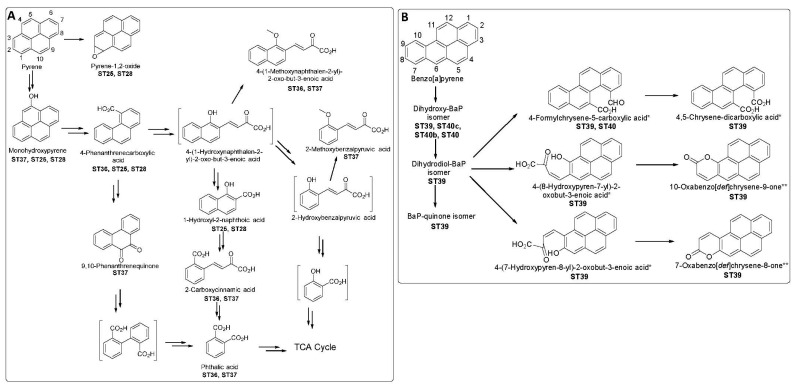
Proposed biodegradation pathways for pyrene (**A**) and benzo[a]pyrene (BaP) (**B**) by bacterial strains isolated from the KSA. ST25: *Stenotrophomonas maltophilia* AJH1; ST28: Consortium of *Pseudomonas aeruginosa* CEES1 and *Bacillus thermosaudia* CEES2; ST36: *Halomonas shengliensis;* ST37: *Achromobacter xylosoxidans;* ST39: *Staphylococcus haemoliticus* 10SBZ1A; ST40a: *Bradyrhizobium japonicun* JBZ1A, ST40b: *Micrococcus luteus* JBZ2B, ST40c: *Bacillus cereus* JBZ5E; ST40: consortium of ST40a, ST40b, and ST40c. ST refers to the research study as indicated in Table 1. The small letters refer to a specific strain (for instance ST40a refers to ST40, but is related to the strain *Bradyrhizobium japonicun* JBZ1A).

In the case of pyrene degradation by *Halomonas shengliensis* and *Halomonas smyrnensis* (ST36), several metabolites were identified, including 4-phenanthrenecarboxylic acid, 4-(1-hydroxynaphthalen-2-yl)-2-oxo-but-3-enoic acid, and phthalic acid. Likewise, the degradation of pyrene by *Achromobacter xylosoxidans* PY4 resulted in the identification of monohydroxypyrene, 4-(1-methoxynaphthalen-2-yl)-2-oxo-but-3-enoic acid, 9,10-phenanthrenequinone, 2-methoxybenzalpyruvic acid, and dibutyl phthalate (ST37). These identified metabolites in the metabolic pathway of pyrene in ST36 and ST37 suggest that pyrene oxidation takes place at C4 and C5 positions, followed by ring cleavage at these positions (Figure 8). 4-Phenanthrenecarboxylic acid was also observed in pyrene degradation using *S. maltophilia* strain AJH1 (ST25), supporting the C4-C5 ring cleavage of pyrene. This metabolite was also observed using a consortium of *P. aeruginosa* CEES1 and *B. thermosaudia* CEES2 (ST28). However, the identification of pyrene-1,2-oxide and 1-hydroxypyrene in the degradation of pyrene in studies ST25 and TS28 suggests a ring cleavage at C1 and C2, but no further phenalene-based metabolites were identified. It is not uncommon for a single strain to follow more than one pathway in the degradation of complex PAHs. A tentative metabolic pathway for pyrene by bacterial strains isolated from the KSA is shown in Figure 8 (ST25, ST28, ST36, ST37)

In relation to the degradation of benzo[a]pyrene using *Staphylococcus haemoliticus* 10SBZ1A (ST39), the metabolites dihydroxy-benzo[a]pyrene and benzo[a]pyrene-quinone were observed. However, the specific isomer was not identified. Additionally, either 4,5-chrysene-dicarboxylic acid, 4-(8-hydroxypyren-7-yl)-2-oxobut-3-enoic acid, or 4-(7-hydroxypyren-8-yl)-2-oxobut-3-enoic acid was observed, and either 10-oxabenzo[def]chrysene-9-one or 7-oxabenzo[def]chrysene-8-one was also observed. The structural similarity of these metabolites, their exact molar masses, and the lack of standard samples for comparison restricted the proper identification of these metabolites. Additionally, 4-formylchrysene-5-carboxylic was also observed in the degradation of benzo[a]pyrene in study ST39, indicating a ring cleavage at C4-C5 of benzo[a]pyrene. Dihydroxy-benzo[a]pyrene, methylated-dihydrodiol-benzo[a]pyrene, and 4-formylchrysene-5-carboxylic acid were also detected when using individual and consortium of *Bradyrhizobium japonicun* JBZ1A, *Micrococcus luteus* JBZ2B, and *Bacillus cereus* JBZ5E (ST40). A tentative degradation pathway is shown for benzo[a]pyrene in Figure 8 for ST39 and ST40, although no early metabolites (hydroxylated form of benzo[a]pyrene) were identified. 

These findings highlight the complexity of biodegradation of PAHs and the diverse metabolites produced during their degradation by various microbial strains. A detailed understanding of the biodegradation pathways of these PAHs will provide crucial insights for advancing future bioremediation strategies and discovering novel enzymes with unique functions. However, the limited number of studies on PAH biodegradation constrains our understanding of the complete metabolic pathways; therefore, there is a need for further research on this topic to elucidate the biodegradation pathways for PAHs. 

## 4. Knowledge Gaps

As discussed earlier, many studies have been dedicated to the biodegradation of crude oil products, mainly PAHs. Various bacterial strains have been isolated and characterized, and metabolites have been identified, and this has contributed to improving our knowledge of this research topic [81]. However, several fields in biodegradation have not received much attention, and they are discussed below.

### 4.1. Studies on Higher PAHs and Complex Oil Products

The aforementioned studies in the review in particular, and those published globally in general, have given limited attention to PAHs with more than five fused rings. For instance, coronene, a PAH with seven fused rings, has been reported in only two studies, one of which was conducted in the KSA [63,83]. However, no studies have focused on metabolite identification or biotechnical degradation pathways for this compound. Furthermore, no investigations have been carried out on the biodegradation of complex PAHs with more than seven rings. 

As stated earlier, in addition to saturates and mono- and polycyclic aromatic hydrocarbons, oil products contain resins and asphaltenes [4]. Relatively little research has been conducted on the biodegradation of these components, in contrast to the attention given to saturates and aromatics due to the high complexity of the structures of asphaltenes and resins [4]. Evidence of bacterial degradation of asphaltenes and resins has been reported in various parts of the world, although this degradation is only partial since it is primarily associated with aliphatic moieties only of these compounds [4]. However, so far, there is no report on the KSA’s biodegradation of resins and asphaltenes. Thus, the bacterial degradation of these oil components awaits elucidation in the KSA.

### 4.2. Omics Studies: Whole-Genome Analysis, Transcriptomics or Proteomics

Genetic studies involving gene expression, transcriptomics, proteomics, or whole genome analysis have been carried out on bacterial degradation of MAHs and PAHs [5]. From these studies, various genes encoding enzymes that degrade aromatic compounds have been identified, and their roles clarified; in addition, some of these genes have been used in genetic engineering to generate bacterial strains with high capabilities to degrade PAHs. For instance, the genes coding for dioxygenase (the first enzyme in MAH and PAH degradation) from *Mycobacterium* sp. PYR-1 was expressed in an *Escherichia coli* strain, enabling this strain to degrade PAHs [91]. A recombinant strain *Pseudomonas fluorescens* HK44 and *Pseudomonas putida* KT2442 were constructed with naphthalene-catabolic plasmids [92,93,94]. These genes have been used as probes to detect bacteria that degrade PAHs in various environments [95,96,97].

However, in the KSA, this area of research has received little attention. To date, only two studies have focused on “omics” analysis in the context of petroleum product biodegradation. In the first study, a whole-genome analysis of a bacterial strain, *Idiomarina piscisalsi* 10PY1A, that degrades pyrene was carried out. The results revealed the existence of several open-reading frames of putative enzymes that degrade aromatic compounds. These included Rieske non-heme iron aromatic ring-hydroxylating oxygenase, homogentisate 1,2-dioxygenase, 1-hydroxy-2-naphthoate hydroxylase, and phthalate 3,4-dioxygenase, among others [98]. In the second investigation (ST37), proteomic analysis of *Achromobacter xylosoxidans* PY4 was carried out in the context of pyrene degradation, and gene products of the lower pyrene degradation pathway were identified, including 4-hydroxyphenylpyruvate dioxygenase and homogentisate 1,2-dioxygenase, the latter being an enzyme involved in ring opening. 

These two “omics” studies have permitted the identification of enzymes of lower PAH degradation pathways only. Thus, information on the upper PAH degradation pathways remains to be elucidated. In addition, no reports exist on omics investigations of PAHs with more than four rings, such as benzo[a]pyrene or coronene, in bacteria isolated in the KSA. 

### 4.3. The Potential of Fungi and Algae in the Degradation of Petroleum Products

As discussed earlier, this review has focused only on the bacterial strains degrading petroleum products (as per the inclusion criteria). However, the degradation of petroleum products by fungal strains has also been reported in other parts of the world [99,100,101]. In the KSA, a fungal strain, *Hortaea* sp. B15, was shown to degrade phenanthrene, chrysene, and pyrene [102,103]. In another study (ST33), a fungal strain *Cryptococcus* s.p MR 22 was isolated from contaminated samples (along with the bacterial stain *Holomonas* sp. BR04), and found to degrade phenanthrene and anthracene [55]. This is in line with studies carried out in other parts of the world indicating the potential of fungi in the removal of petroleum pollutants [104].

Likewise, several reports have shown that microalgal organisms can degrade various petroleum products, including PAH [105,106,107]. These organisms are important since they are photosynthetic, using CO_2_ to synthesize organic molecules, thus contributing to the removal of CO_2_ from the environment. In addition, these organisms are associated with growth and biomass accumulation capability, great diversity, and robust adaptability in various environments [108]. In the KSA, so far, only one study has been dedicated to the use of microalgae in petroleum product degradation [109]. This study, carried out by our research team, involved using a *Gonium pectorale* strain to degrade phenanthrene and anthracene [109]. In other parts of the world, reports indicate that complex PAH, such as pyrene and benzo[a]pyrene, can efficiently be degraded by microalgal strains [105,106,107].

The use of the microalgae–bacteria consortium is emerging as a new strategy for pollutant removal [105,108,110]. On the one hand, algae produce O_2_ through photosynthesis, which supports aerobic bacterial activity, crucial for breaking down organic pollutants. On the other hand, bacteria produce CO_2_ that algae will utilize in the photosynthetic process. In addition, algae can absorb higher concentrations of pollutants with less negative effect on their growth compared to bacteria, thus allowing these bacteria to grow actively in environments contaminated with high concentrations of pollutants. This mutualistic relationship improves the overall effectiveness and rate of pollutant removal [105]. This concept awaits investigation in the context of cleaning the environment in the KSA. 

### 4.4. Biodegradation Stimulation Methods

Several reports have been dedicated to increasing biodegradation by either adding nutrients, a process known as biostimulation, or external biosurfactants. 

This increase in bacterial growth can lead to a higher degradation rate of organic pollutants, including hydrocarbons. The beneficial effects of biostimulation have been demonstrated in the removal of hydrocarbons in various studies conducted in both soil and marine environments [12,111,112]. However, the application of biostimulation for pollutant removal has not yet been explored in Saudi Arabia.

Another strategy to increase the efficiency of biodegradation is the use of the surfactant. They are surface-active agents that reduce the surface tension between water and hydrophobic pollutants (such as PHAs), thus enhancing pollutants’ solubility and availability, making them more accessible to microorganisms. As stated earlier, the active bacteria degrading petroleum products express surfactants (known as biosurfactants) to increase the solution of the pollutants [28]. Therefore, biodegradation or bioremediation can be facilitated by adding surfactants to the environment, thereby enhancing both pollutant solubility and degradation, a strategy that has been evaluated and tested already [113,114] but awaits evaluation in the context oil contaminated environments, including those of Saudi Arabia. 

### 4.5. Degradation of Petroleum Products in Anaerobic Conditions

The biodegradation of pollutants is primarily carried out under aerobic conditions because oxygen serves as an electron acceptor, enabling faster and more efficient breakdown of complex organic pollutants by microorganisms. As discussed earlier, aerobic microbes have well-established pathways, primarily involving oxygenases, that directly facilitate the degradation of hydrocarbons. In contrast, anaerobic conditions present challenges due to the lack of oxygen, which is a key element in many metabolic pathways for degradation. Under anaerobic conditions, microbes must rely on alternative electron acceptors, such as sulfate, carbon dioxide, and nitrate, which leads to slower degradation rates [6]. The anaerobic degradation of petroleum products has been reported, however, to a much lesser extent than in aerobic conditions [6,79,115]. For example, in the case of Saudi Arabia, there have been no reports on the degradation of petroleum products under anaerobic conditions. All studies summarized in this review (see Table 1) were conducted under aerobic conditions. Overall, the metabolic pathways associated with anaerobic degradation are more complex and less well-understood; therefore, exploring these mechanisms could lead to the discovery of new biochemical pathways that may be leveraged in biocatalysis processes. Consequently, further research on anaerobic degradation is essential.

## 5. Conclusions and Future Perspective

This review has highlighted the research carried out so far in the KSA on the microbial degradation of petroleum products. Bacteria belonging to several genera have been identified, isolated, and characterized. Overall, the distribution (in terms of species or genera) and frequency of identification of these bacteria are similar to those reported in other parts of the world. The degradation of PAHs has been documented. However, more efforts need to be dedicated to the degradation of more complex PAHs. Likewise, the use of microalgae, either alone or in combination with bacteria, offers an alternative approach to improve the degradation of recalcitrant PAHs.

Extreme environmental conditions, including dryness, high temperature, and high salinity characterize the KSA. Thus, it is conceivable that bacteria have adapted to this environment by expressing enzymes with unique catabolic activities. Identifying and characterizing such enzymes will offer, for instance, new biocatalytic strategies or contribute to creating genetically engineered microorganisms (GEMs) with unique catalytic properties. Therefore, there is a need for more comprehensive studies on “Omics” on bacterial strains in the KSA.

Finally, the isolated and characterized active bacteria described in this review underscore the necessity of field studies on bioremediation. The concept of bioremediation for the removal of pollutants in natural conditions of contamination has been evaluated in various parts of the world, and some of these products are commercially available [5]. The active bacteria identified in the KSA have the potential to be used in real-world cleaning of oil-contaminated environments, which highlights the promising prospect of applying bioremediation to address environmental challenges. 

## Data Availability

Data is contained within the article or Supplementary Materials.

## Supplementary Materials

The following are available online at https://www.mdpi.com/article/10.3390/toxics12110800/s1, Table S1: Frequency of isolated bacterial strains degrading petroleum products in the Kingdom of Saudi Arabia.

## References

[1] Fakher S., Ahdaya M., Elturki M., Imqam A. (2020). Critical review of asphaltene properties and factors impacting its stability in crude oil. J. Pet. Explor. Prod. Technol..

[2] Chen B., Ye X., Zhang B., Jing L., Lee K., Sheppard C.B.T. (2019). Chapter 22—Marine Oil Spills—Preparedness and Countermeasures. World Seas: An Environmental Evaluation.

[3] Fakhru’l-Razi A., Pendashteh A., Abdullah L.C., Biak D.R.A., Madaeni S.S., Abidin Z.Z. (2009). Review of technologies for oil and gas produced water treatment. J. Hazard. Mater..

[4] Nzila A., Musa M. (2021). Current Knowledge and Future Challenges on Bacterial Degradation of the Highly Complex Petroleum Products Asphaltenes and Resins. Front. Environ. Sci..

[5] Haritash A.K. (2020). A comprehensive review of metabolic and genomic aspects of PAH-degradation. Arch. Microbiol..

[6] Nzila A. (2018). Biodegradation of high-molecular-weight polycyclic aromatic hydrocarbons under anaerobic conditions: Overview of studies, proposed pathways and future perspectives. Environ. Pollut..

[7] Nzila A. (2018). Current status of the degradation of aliphatic and aromatic petroleum hydrocarbons by thermophilic microbes and future perspectives. Int. J. Environ. Res. Public Health.

[8] Sun K., Song Y., He F., Jing M., Tang J., Liu R. (2021). A review of human and animals exposure to polycyclic aromatic hydrocarbons: Health risk and adverse effects, photo-induced toxicity and regulating effect of microplastics. Sci. Total Environ..

[9] Patel A., Shaikh S., Jain K., Desai C., Madamvar D. (2020). Polycyclic Aromatic Hydrocarbons: Sources, Toxicity, and Remediation Approaches. Front. Microbiol..

[10] Ramesh A., Harris K.J., Archibong A.E., Gupta R.C. (2017). Chapter 40—Reproductive Toxicity of Polycyclic Aromatic Hydrocarbons. Reproductive and Developmental Toxicology.

[11] Jesus F., Pereira J.L., Campos I., Santos M., Ré A., Keizer J., Nogueira A., Gonçalves F.J.M., Abrantes N., Serpa D. (2022). A review on polycyclic aromatic hydrocarbons distribution in freshwater ecosystems and their toxicity to benthic fauna. Sci. Total Environ..

[12] Sayed K., Baloo L., Sharma N.K. (2021). Bioremediation of Total Petroleum Hydrocarbons (TPH) by Bioaugmentation and Biostimulation in Water with Floating Oil Spill Containment Booms as Bioreactor Basin. Int. J. Environ. Res. Public Health.

[13] Davoodi S.M., Miri S., Taheran M., Brar S.K., Galvez-Cloutier R., Martel R. (2020). Bioremediation of Unconventional Oil Contaminated Ecosystems under Natural and Assisted Conditions: A Review. Environ. Sci. Technol..

[14] Adedeji J., Tetteh E., Amankwa M., Asante-Sackey D., Frimpong S., Armah E., Rathilal S., Mohammadi A., Manimagalay C. (2022). Microbial Bioremediation and Biodegradation of Petroleum Products—A Mini Review. Appl. Sci..

[15] Michel J., Fingas M. (2011). Chapter 37—1991 Gulf War Oil Spill. Oil Spill Science and Technology.

[16] Marzooq H., Naser H.A., Elkanzi E.M. (2019). Quantifying exposure levels of coastal facilities to oil spills in Bahrain, Arabian Gulf. Environ. Monit. Assess..

[17] Al-Ghouti M.A., Al-Kaabi M.A., Ashfaq M.Y., Da’na D.A. (2019). Produced water characteristics, treatment and reuse: A review. J. Water Process Eng..

[18] El-Sayed A.A.H., Al-Blehed M.S. (1999). Bacterial Isolate from Arabian Gulf Coast Soils in Saudi Arabia Able to Degrade Arab Crude Oil. J. King Saud Univ.-Eng. Sci..

[19] Palleroni N.J., Pieper D.H., Moore E.R.B., Timmis K.N. (2010). Microbiology of Hydrocarbon-Degrading *Pseudomonas*. Handbook of Hydrocarbon and Lipid Microbiology.

[20] Söhngen N.L. (1913). Benzin, petroleum, paraffinöl und paraffin als kohlenstoff-und energiequelle für mikroben. Zentr. Bacteriol. Parasitenk. Abt. II.

[21] van Beilen J.B., Witholt B. (2004). Alkane degradation by pseudomonads. Pseudomonas: Volume 3 Biosynthesis of Macromolecules and Molecular Metabolism.

[22] Jiménez J.I., Miñambres B., García J.L., Díaz E. (2004). Genomic insights in the metabolism of aromatic compounds in *Pseudomonas*. Pseudomonas: Volume 3 Biosynthesis of Macromolecules and Molecular Metabolism.

[23] Pieper D.H., Reineke W. (2004). Degradation of chloroaromatics by Pseudomona(d)s. Pseudomonas: Volume 3 Biosynthesis of Macromolecules and Molecular Metabolism.

[24] Gilani R.A., Rafique M., Rehman A., Munis M.F.H., ur Rehman S., Chaudhary H.J. (2016). Biodegradation of chlorpyrifos by bacterial genus *Pseudomonas*. J. Basic. Microbiol..

[25] Wilkes R.A., Aristilde L. (2017). Degradation and metabolism of synthetic plastics and associated products by *Pseudomonas* sp. capabilities and challenges. J. Appl. Microbiol..

[26] Arora P.K. (2020). Bacilli-Mediated Degradation of Xenobiotic Compounds and Heavy Metals. Front. Bioeng. Biotechnol..

[27] Mishra S., Lin Z., Pang S., Zhang Y., Bhatt P., Chen S. (2021). Biosurfactant is a powerful tool for the bioremediation of heavy metals from contaminated soils. J. Hazard. Mater..

[28] Eras-Muñoz E., Farré A., Sánchez A., Font X., Gea T. (2022). Microbial biosurfactants: A review of recent environmental applications. Bioengineered.

[29] Rache-Arce D., Machacado-Salas M., Rosero-García D. (2022). Hydrocarbon-degrading bacteria in Colombia: Systematic review. Biodegradation.

[30] Christine M. (2004). Microbial Diversity Unbound: What DNA-based techniques are revealing about the planet’s hidden biodiversity. Bioscience.

[31] Wade W. (2002). Unculturable Bacteria—The Uncharacterized organisms that Cause Oral Infections. J. R. Soc. Med..

[32] Gu J.-D. (2021). On Enrichment Culturing and Transferring Technique. Appl. Environ. Biotechnol..

[33] El Hanafy A.A.E.M., Anwar Y., Mohamed S.A., Al-Garni S.M.S., Sabir J.S.M., Abuzinadah O.A., Al Mehdar H., Alfaidi A.W., Ahmed M.M.M. (2016). Isolation and identification of bacterial consortia responsible for degrading oil spills from the coastal area of Yanbu, Saudi Arabia. Biotechnol. Biotechnol. Equip..

[34] El-Rab S.M.F.G., Hassan A.M., Abdelmigid H.M. (2016). Evaluation of genotoxicity and mutagenicity induced by crude oil contaminated water before and after biodegradation. Res. J. Pharm. Biol. Chem. Sci..

[35] Al-Dhabaan F.A. (2019). Morphological, biochemical and molecular identification of petroleum hydrocarbons biodegradation bacteria isolated from oil polluted soil in Dhahran, Saud Arabia. Saudi J. Biol. Sci..

[36] Afkar E., Hafez A., Ibrahim R., Aldayel M. (2021). Effective Removal of Alkanes and Polycyclic Aromatic Hydrocarbons by Bacteria from Soil Chronically Exposed to Crude Petroleum Oil. Appl. Ecol. Environ. Res..

[37] Hazaimeh M., Kanaan B.M., AlFaleh F.A., Elhaig M.M., Khamaiseh E.I., Zia Q., Alaidarous M., Seth C.S., Alsowayeh N., Ahmad F. (2024). Biodegradation of petroleum hydrocarbons using a novel bacterial strain isolated from hydrocarbons contaminated soil of Saudi Arabia. Biocatal. Agric. Biotechnol..

[38] Abdel-Megeed A., Al-Harbi N., Al-Deyab S. (2010). Hexadecane degradation by bacterial strains isolated from contaminated soils. Afr. J. Biotechnol..

[39] Le T.N., Mikolasch A., Awe S., Sheikhany H., Klenk H.P., Schauer F. (2010). Oxidation of aliphatic, branched chain, and aromatic hydrocarbons by Nocardia cyriacigeorgica isolated from oil-polluted sand samples collected in the Saudi Arabian Desert. J. Basic. Microbiol..

[40] El Tarrs A.E., Shahaby A.F., Awad N.S., Bahobial A.S., El Abib O.A. (2012). In vitro screening for oil degrading bacteria and evaluation of their biodegradation potential for hydrocarbon. Afr. J. Microbiol. Res..

[41] Chandrasekaran S., Pugazhendi A., Banu R.J., Ismail I.M.I., Qari H.A. (2018). Biodegradation of phenol by a moderately halophilic bacterial consortium. Environ. Prog. Sustain. Energy.

[42] Arafa M.A. (2003). Biodegradation of Some Aromatic Hydrocarbons (BTEXs) by a Bacterial Consortium Isolated from Polluted Site in Saudi Arabia. Pak. J. Biol. Sci..

[43] Nzila A., Thukair A., Sankara S., Chanbasha B., Musa M.M.M.M. (2016). Isolation and characterization of naphthalene biodegrading *Methylobacterium radiotolerans* bacterium from the eastern coastline of the Kingdom of Saudi Arabia. Arch. Environ. Prot..

[44] Farraj D., Alkufeidy R., Alkubaisi N., Alshammari M. (2020). Polynuclear aromatic anthracene biodegradation by psychrophilic Sphingomonas sp., cultivated with tween-80. Chemosphere.

[45] Hesham A.E.L., Alrumman S.A., Al-Amari J.A. (2016). 16S rDNA Phylogenetic and RAPD–PCR Analyses of Petroleum Polycyclic Aromatic Hydrocarbons-Degrading Bacteria Enriched from Oil-Polluted Soils. Arab. J. Sci. Eng..

[46] Amran R.H., Jamal M.T., Pugazhendi A., Al- Harbi M., Bowrji S. (2022). Petroleum Hydrocarbon Degradation and Treatment of Automobile Service Station Wastewater by Halophilic Consortia Under Saline Conditions. Nat. Environ. Pollut. Technol..

[47] Arulazhagan P., Al-Shekri K., Huda Q., Godon J.J., Basahi J.M., Jeyakumar D. (2017). Biodegradation of polycyclic aromatic hydrocarbons by an acidophilic Stenotrophomonas maltophilia strain AJH1 isolated from a mineral mining site in Saudi Arabia. Extremophiles.

[48] Oyehan T.A., Al-Thukair A.A. (2017). Isolation and characterization of PAH-degrading bacteria from the Eastern Province, Saudi Arabia. Mar. Pollut. Bull..

[49] Pugazhendi A., Qari H., Al-Badry Basahi J.M., Godon J.J., Dhavamani J. (2017). Role of a halothermophilic bacterial consortium for the biodegradation of PAHs and the treatment of petroleum wastewater at extreme conditions. Int. Biodeterior. Biodegrad..

[50] Jamal M., Pugazhendi A. (2018). Degradation of petroleum hydrocarbons and treatment of refinery wastewater under saline condition by a halophilic bacterial consortium enriched from marine environment (Red Sea), Jeddah, Saudi Arabia. 3Biotech.

[51] Jamal M. (2020). Enrichment of Potential Halophilic Marinobacter Consortium for *Mineralization* of Petroleum Hydrocarbons and Also as Oil Reservoir Indicator in Red Sea, Saudi Arabia. Polycycl. Aromat. Compd..

[52] Budiyanto F., Thukair A., Al-Momani M., Musa M.M., Nzila A. (2018). Characterization of Halophilic Bacteria Capable of Efficiently Biodegrading the High-Molecular-Weight Polycyclic Aromatic Hydrocarbon Pyrene. Environ. Eng. Sci..

[53] Alrumman S., Hesham A.E.-L., Alamri S. (2016). Isolation, fingerprinting and genetic identification of indigenous PAHs degrading bacteria from oil-polluted soils. J. Environ. Biol..

[54] Nzila A., Ramirez C.O., Musa M.M., Sankara S., Basheer C., Li Q.X. (2018). Pyrene biodegradation and proteomic analysis in Achromobacter xylosoxidans, PY4 strain. Int. Biodeterior. Biodegrad..

[55] Al Farraj D.A., Hadibarata T., Yuniarto A., Alkufeidy R.M., Alshammari M.K., Syafiuddin A. (2020). Exploring the potential of halotolerant bacteria for biodegradation of polycyclic aromatic hydrocarbon. Bioprocess. Biosyst. Eng..

[56] Al-Mur B.A., Pugazhendi A., Jamal M.T. (2021). Application of integrated extremophilic (halo-alkalo-thermophilic) bacterial consortium in the degradation of petroleum hydrocarbons and treatment of petroleum refinery wastewater under extreme condition. J. Hazard. Mater..

[57] Al-Thukair A.A., Malik K. (2016). Pyrene metabolism by the novel bacterial strains Burkholderia fungorum (T3A13001) and Caulobacter sp (T2A12002) isolated from an oil-polluted site in the Arabian Gulf. Int. Biodeterior. Biodegrad..

[58] Nzila A., Sankara S., Al-Momani M., Musa Musa M., Musa M.M. (2017). Isolation and characterisation of bacteria degrading polycyclic aromatic hydrocarbons: Phenanthrene and anthracene. Arch. Environ. Prot..

[59] Alfaify A., Mir M., Alrumman S. (2022). Klebsiella oxytoca: An efficient pyrene-degrading bacterial strain isolated from petroleum-contaminated soil. Arch. Microbiol..

[60] Nzila A., Musa M.M., Sankara S., Al-Momani M., Xiang L., Li Q.X. (2021). Degradation of benzo[a]pyrene by halophilic bacterial strain Staphylococcus haemoliticus strain 10SBZ1A. PLoS ONE.

[61] Nzila A., Musa M.M., Afuecheta E., Al-Thukair A., Sankaran S., Xiang L., Li Q.X. (2023). Benzo[A]Pyrene Biodegradation by Multiple and Individual Mesophilic Bacteria under Axenic Conditions and in Soil Samples. Int. J. Environ. Res. Public Health.

[62] Al-Thukair A.A., Malik K., Nzila A. (2020). Biodegradation of selected hydrocarbons by novel bacterial strains isolated from contaminated Arabian Gulf sediment. Sci. Rep..

[63] Okeyode A.H., Al-Thukair A., Chanbasha B., Nazal M.K., Afuecheta E., Musa M.M., Algarni S., Nzila A. (2023). Degradation of the highly complex polycyclic aromatic hydrocarbon coronene by the halophilic bacterial strain Halomonas caseinilytica, 10SCRN4D. Arch. Environ. Prot..

[64] Shahaby A.F., Alharthi A.A., El Tarras A.E. (2015). Bioremediation of Petroleum Oil by Potential Biosurfactant-Producing Bacteria using Gravimetric Assay. Int. J. Curr. Microbiol. App. Sci..

[65] Pugazhendi A., Abbad Wazin H., Qari H., Basahi J.M.A.B., Godon J.J., Dhavamani J. (2017). Biodegradation of low and high molecular weight hydrocarbons in petroleum refinery wastewater by a thermophilic bacterial consortium. Environ. Technol..

[66] Ibrahim M.M., Al-Turki A., Al-Sewedi D., Arif I.A., El-Gaaly G.A. (2015). Molecular application for identification of polycyclic aromatic hydrocarbons degrading bacteria (PAHD) species isolated from oil polluted soil in Dammam, Saud Arabia. Saudi J. Biol. Sci..

[67] Eman A.H.M., Naeima M.H.Y., Azza G. (2012). F Isolation and molecular identification of polyaromatic hydrocarbons- utilizing bacteria from crude petroleum oil samples. Afr. J. Microbiol. Res..

[68] Bahobail A., Gad El-Rab S.M.F., Amin G.A. (2016). Locally Isolated Bacterial Strains with Multiple Degradation Potential Capabilities on Petroleum Hydrocarbon Pollutants. Adv. Microbiol..

[69] Alghamdi A., El-bendary M., Alabdalall A., Ababutain I. (2017). Petroleum oil biodegradation potential of some isolated bacteria from Saudi Arabia. J. Food Agric. Environ..

[70] Anwar Y., El-Hanafy A.A., Sabir J.S.M., Al-Garni S.M.S., Al-Ghamdi K., Almehdar H., Waqas M. (2017). Characterization of Mesophilic Bacteria Degrading Crude Oil from Different Sites of Aramco, Saudi Arabia. Polycycl. Aromat. Compd..

[71] Al-Dhabi N.A., Esmail G.A., Arasu M.V. (2020). Enhanced production of biosurfactant from bacillus subtilis strain al-dhabi-130 under solid-state fermentation using date molasses from saudi arabia for bioremediation of crude-oil-contaminated soils. Int. J. Environ. Res. Public Health.

[72] Ameen F., Al-Homaidan A.A. (2023). Oily bilge water treatment using indigenous soil bacteria: Implications for recycling the treated sludge in vegetable farming. Chemosphere.

[73] Yaman C. (2020). Performance and Kinetics of Bioaugmentation, Biostimulation, and Natural Attenuation Processes for Bioremediation of Crude Oil-Contaminated Soils. Processes.

[74] Róźalska S., Iwanicka-Nowicka R., Długoński J. (2016). Organic Pollutants Degradation by Microorganisms: Genomics, Metagenomics and Metatranstriptomics Backgrounds. Microbial Biodegradation: From Omics to Function and Application.

[75] Hidalgo K.J., Sierra-Garcia I.N., Dellagnezze B.M., de Oliveira V.M. (2020). Metagenomic Insights into the Mechanisms for Biodegradation of Polycyclic Aromatic Hydrocarbons in the Oil Supply Chain. Front. Microbiol..

[76] Abed R.M.M., Al-Thukair A., De Beer D. (2006). Bacterial diversity of a cyanobacterial mat degrading petroleum compounds at elevated salinities and temperatures. FEMS Microbiol. Ecol..

[77] An D., Caffrey S.M., Soh J., Agrawal A., Brown D., Budwill K., Dong X., Dunfield P.F., Foght J., Gieg L.M. (2013). Metagenomics of Hydrocarbon Resource Environments Indicates Aerobic Taxa and Genes to be Unexpectedly Common. Environ. Sci. Technol..

[78] Abbasian F., Lockington R., Mallavarapu M., Naidu R. (2015). A Comprehensive Review of Aliphatic Hydrocarbon Biodegradation by Bacteria. Appl. Biochem. Biotechnol..

[79] Dhar K., Subashchandrabose S.R., Venkateswarlu K., Krishnan K., Megharaj M. (2020). Anaerobic Microbial Degradation of Polycyclic Aromatic Hydrocarbons: A Comprehensive Review. Rev. Environ. Contam. Toxicol..

[80] Ghosal D., Ghosh S., Dutta T.K., Ahn Y. (2016). Current State of Knowledge in Microbial Degradation of Polycyclic Aromatic Hydrocarbons (PAHs): A Review. Front. Microbiol..

[81] Méndez García M., García de Llasera M.P. (2021). A review on the enzymes and metabolites identified by mass spectrometry from bacteria and microalgae involved in the degradation of high molecular weight PAHs. Sci. Total Environ..

[82] Nzila A., Musa M.M. (2020). Current Status of and Future Perspectives in Bacterial Degradation of Benzo[a]pyrene. Int. J. Environ. Res. Public Health.

[83] Juhasz A.L., Britz M.L., Stanley G.A. (1997). Degradation of benzo[a]pyrene, dibenz[a,h]anthracene and coronene by Burkholderia cepacia. Water Sci. Technol..

[84] He X., Zheng X., You Y., Zhang S., Zhao B., Wang X., Huang G., Chen T., Cao Y., He L. (2022). Comprehensive chemical characterization of gaseous I/SVOC emissions from heavy-duty diesel vehicles using two-dimensional gas chromatography time-of-flight mass spectrometry. Environ. Pollut..

[85] Tse C., Ma K., Rampelotto P.H. (2016). Growth and Metabolism of Extremophilic Microorganisms. Biotechnology of Extremophiles: Advances and Challenges.

[86] Fathepure B.Z. (2014). Recent studies in microbial degradation of petroleum hydrocarbons in hypersaline environments. Front. Microbiol..

[87] Arulazhagan P., Vasudevan N. (2011). Biodegradation of polycyclic aromatic hydrocarbons by a halotolerant bacterial strain *Ochrobactrum* sp. VA1. Mar. Pollut. Bull..

[88] Wu C., Li F., Yi S., Ge F. (2021). Genetically engineered microbial remediation of soils co-contaminated by heavy metals and polycyclic aromatic hydrocarbons: Advances and ecological risk assessment. J. Environ. Manag..

[89] Xiang L., Li G., Wen L., Su C., Liu Y., Tang H., Dai J. (2021). Biodegradation of aromatic pollutants meets synthetic biology. Synth. Syst. Biotechnol..

[90] Seo J., Keum Y., Li Q.X. (2009). Bacterial Degradation of Aromatic Compounds. Int. J. Environ. Res. Public Health.

[91] Khan A.A., Wang R.F., Cao W.W., Doerge D.R., Wennerstrom D., Cerniglia C.E. (2001). Molecular cloning, nucleotide sequence, and expression of genes encoding a polycyclic aromatic ring dioxygenase from *Mycobacterium* sp. strain PYR-1. Appl. Environ. Microbiol..

[92] Ripp S., Nivens D., Ahn Y., Werner C., Jarrell Easter J., Cox C., Burlage R., Sayler G. (2000). Controlled Field Release of a Bioluminescent Genetically Engineered Microorganism for Bioremediation Process Monitoring and Control. Environ. Sci. Technol..

[93] Filonov A.E., Akhmetov L.I., Puntus I.F., Esikova T.Z., Gafarov A.B., Izmalkova TYu Sokolov S.L., Kosheleva I.A., Boronin A.M. (2005). The Construction and Monitoring of Genetically Tagged, Plasmid-Containing, Naphthalene-Degrading Strains in Soil. Microbiology.

[94] Sayler G., Ripp S. (2000). Field applications of genetically engineered microorganisms for bioremediation processes. Curr. Opin. Biotechnol..

[95] Szewczyk R., Kowalski K., Długoński J. (2016). Metabolomics and Crucial Enzymes in Microbial Degradation of Contaminants. Microbial Biodegradation: From Omics to Function and Application.

[96] Debruyn J.M., Chewning C.S., Sayler G.S. (2007). Comparative quantitative prevalence of Mycobacteria and functionally abundant nidA, nahAc, and nagAc dioxygenase genes in coal tar contaminated sediments. Environ. Sci. Technol..

[97] DeBruyn J.M., Mead T.J., Sayler G.S. (2012). Horizontal transfer of PAH catabolism genes in *Mycobacterium*: Evidence from comparative genomics and isolated pyrene-degrading bacteria. Environ. Sci. Technol..

[98] Nzila A., Jung B.K., Kim M.-C., Ibal J.C., Budiyanto F., Musa M.M., Thukair A., Kim S.-J., Shin J.-H. (2018). Complete genome sequence of the polycyclic aromatic hydrocarbons biodegrading bacterium Idiomarina piscisalsi strain 10PY1A isolated from oil-contaminated soil. Korean J. Microbiol..

[99] Hassanshahian M., Amirinejad N., Behzadi M. (2020). Crude oil pollution and biodegradation at the Persian Gulf: A comprehensive and review study. J. Environ. Health Sci. Eng..

[100] Torres-Farradá G., Thijs S., Rineau F., Guerra G., Vangronsveld J. (2024). White Rot Fungi as Tools for the Bioremediation of Xenobiotics: A Review. J. Fungi.

[101] da Silva A.F., Banat I.M., Robl D., Giachini A.J. (2023). Fungal bioproducts for petroleum hydrocarbons and toxic metals remediation: Recent advances and emerging technologies. Bioprocess. Biosyst. Eng..

[102] Al Farraj D.A., Hadibarata T., Yuniarto A., Syafiuddin A., Surtikanti H.K., Elshikh M.S., Al Khulaifi M.M., Al-Kufaidy R. (2019). Characterization of pyrene and chrysene degradation by halophilic *Hortaea* sp. B15. Bioprocess Biosyst. Eng..

[103] Ayu R., Hadibarata T., Farraj D., Elshikh M., Alkufeidy R. (2018). Biodegradation Mechanism of Phenanthrene by Halophilic *Hortaea* sp. B15. Water Air Soil Pollut..

[104] Cerniglia C.E., Sutherland J.B., Timmis K.N. (2010). Degradation of Polycyclic Aromatic Hydrocarbons by Fungi. Handbook of Hydrocarbon and Lipid Microbiology.

[105] Ahmad I. (2022). Microalgae–Bacteria Consortia: A Review on the Degradation of Polycyclic Aromatic Hydrocarbons (PAHs). Arab. J. Sci. Eng..

[106] Dell’ Anno F., Rastelli E., Sansone C., Brunet C., Ianora A., Dell’ Anno A. (2021). Bacteria, Fungi and Microalgae for the Bioremediation of Marine Sediments Contaminated by Petroleum Hydrocarbons in the Omics Era. Microorganisms.

[107] Satpati G.G., Gupta S., Biswas R.K., Choudhury A.K., Kim J.-W., Davoodbasha M. (2023). Microalgae mediated bioremediation of polycyclic aromatic hydrocarbons: Strategies, advancement and regulations. Chemosphere.

[108] Abate R., Oon Y.-S., Oon Y.-L., Bi Y. (2024). Microalgae-Bacteria Nexus for Environmental Remediation and Renewable Energy Resources: Advances, Mechanisms and Biotechnological Applications. Heliyon.

[109] Hoque M.Z., Alqahtani A., Sankaran S., Anand D., Musa M.M., Nzila A., Guerriero G., Siddiqui K.S., Ahmad I. (2023). Enhanced biodegradation of phenanthrene and anthracene using a microalgal-bacterial consortium. Front. Microbiol..

[110] Guo W., Ren H., Jin Y., Chai Z., Liu B. (2024). The bioremediation of the typical persistent organic pollutants (POPs) by microalgae-bacteria consortia: A systematic review. Chemosphere.

[111] Huang Y., Zhou Z., Cai Y., Li X., Huang Y., Hou J., Liu W. (2024). Response of petroleum-contaminated soil to chemical oxidation combined with biostimulation. Ecotoxicol. Environ. Saf..

[112] Adams G.O., Fufeyin P.T., Okoro S.E., Ehinomen I. (2015). Bioremediation, Biostimulation and Bioaugmention: A Review. Int. J. Environ. Bioremediat Biodegrad..

[113] Ambust S., Das A.J., Kumar R. (2021). Bioremediation of petroleum contaminated soil through biosurfactant and *Pseudomonas* sp. SA3 amended design treatments. Curr. Res. Microb. Sci..

[114] Ng Y.J., Lim H.R., Khoo K.S., Chew K.W., Chan D.J.C., Bilal M., Munawaroh H.S.H., Show P.L. (2022). Recent advances of biosurfactant for waste and pollution bioremediation: Substitutions of petroleum-based surfactants. Environ. Res..

[115] Wartell B., Boufadel M., Rodriguez-Freire L. (2021). An effort to understand and improve the anaerobic biodegradation of petroleum hydrocarbons: A literature review. Int. Biodeterior. Biodegrad..

